# Quantitative autistic traits ascertained in a national survey of 22 529 Japanese schoolchildren

**DOI:** 10.1111/acps.12034

**Published:** 2012-11-22

**Authors:** Y Kamio, N Inada, A Moriwaki, M Kuroda, T Koyama, H Tsujii, Y Kawakubo, H Kuwabara, K J Tsuchiya, Y Uno, J N Constantino

**Affiliations:** 1 Department of Child and Adolescent Mental Health, National Institute of Mental Health, National Center of Neurology and PsychiatryTokyo, Japan; 2 Department of Child Neuropsychiatry, Graduate School of Medicine, University of TokyoTokyo, Japan; 3 Research Center for Child Mental Development, United Graduate School of Child Development, School of Medicine, Hamamatsu UniversityHamamatsu, Japan; 4 Department of Psychiatry and Psychiatry for Parents and Children, Graduate School of Medicine, Nagoya UniversityNagoya, Japan; 5 Departments of Psychiatry and Pediatrics, School of Medicine, Washington UniversitySt. Louis, MO, USA

**Keywords:** autism, questionnaire, prevalence, classification, diagnosis

## Abstract

**Objective:**

Recent epidemiologic studies worldwide have documented a rise in prevalence rates for autism spectrum disorders (ASD). Broadening of diagnostic criteria for ASD may be a major contributor to the rise in prevalence, particularly if superimposed on an underlying continuous distribution of autistic traits. This study sought to determine the nature of the population distribution of autistic traits using a quantitative trait measure in a large national population sample of children.

**Method:**

The Japanese version of the Social Responsiveness Scale (SRS) was completed by parents on a nationally representative sample of 22 529 children, age 6–15.

**Results:**

Social Responsiveness Scale scores exhibited a skewed normal distribution in the Japanese population with a single-factor structure and no significant relation to IQ within the normal intellectual range. There was no evidence of a natural ‘cutoff’ that would differentiate populations of categorically affected children from unaffected children.

**Conclusion:**

This study provides evidence of the continuous nature of autistic symptoms measured by the SRS, a validated quantitative trait measure. The findings reveal how paradigms for diagnosis that rest on arbitrarily imposed categorical cutoffs can result in substantial variation in prevalence estimation, especially when measurements used for case assignment are not standardized for a given population.

**Significant outcomes**

- In a large Japanese child population, behaviorally measured autistic traits are continuously distributed without any apparent deflection in the distribution plot that would signal a natural cutoff for categorical diagnoses. This is similar to the distribution pattern in US and European samples.
- Autistic traits measured quantitatively by parents differ slightly by culture, suggesting the need to interpret autism spectrum disorder (ASD) severity ratings with the use of culturally calibrated norms.
- Many children who do not meet the diagnosis of ASD exhibit elevations in autistic traits measured quantitatively, suggesting the need to reconsider current diagnostic systems that assume discontinuity between affected and unaffected populations.

**Limitations**

- The response rate of this nationwide survey was 29%.
- There is a possibility of bias that would differentiate respondents vs. non-respondents.
- High-scoring children in the sample as a whole were not confirmed using diagnostic instruments, although quantitatively measured autistic traits were extensively clinically confirmed for a separate smaller sample.

## Introduction

Although to date the designation of pervasive developmental disorders in children – and the services to which affected children are entitled – rest on categorical case definitions, the concept of an autistic *spectrum*, along which the number and intensity of autistic features vary continuously from mild to severe, dates back to early epidemiological research by Wing and Gould 1. Wing 2 subsequently developed the concept of the autistic continuum, broadening the case designation beyond classic autism to encompass the mildest (but most prevalent) of the autism spectrum disorders (ASDs), pervasive developmental disorder not otherwise specified (PDD-NOS) assigned by diagnostic and statistical manual of mental disorders: text revision (DSM-IV-TR) 3. Several lines of subsequent research 4–7 now strongly suggest that the autism spectrum extends beyond this PDD-NOS subcategory to include subclinical levels of symptomatology, which are known to aggregate in the undiagnosed members of families with multiple-incidence autism. Very recently, Lord et al. 8 observed that diagnostic assignments of autistic disorder, Asperger's disorder, and PDD-NOS made by expert clinicians varied considerably across sites, despite the fact that distributions of scores on validated measures were similar. They concluded that current taxonomies should be revised to place priority on characterizing the dimensions of ASD while controlling for IQ and language level.

Clarifying the nature of the population distribution of autistic traits and symptoms across cultures has substantial implications for understanding a rise in prevalence over time 9 and for establishing the ‘boundaries’ of clinical affectation. A recent Korean study 10 suggested the highest ever reported prevalence for categorically defined ASD in a total population sample; in that study, symptom counts were found to be continuously distributed in the population.

### Aims of the study

This study determined whether autistic traits would be continuously distributed in a population-based sample to establish the appropriate epidemiologic framework for interpreting the rise in estimated autism spectrum disorders prevalence over time.

## Material and methods

### Participants

The participants comprised a normative sample (*n* = 22 529) of schoolchildren, a child psychiatric clinical sample (*n* = 417), and typically developing (TD) children (*n* = 61). The normative sample was exclusively assessed using the Japanese version of the Social Responsiveness Scale (SRS) 11. The latter two samples were more extensively assessed using standard diagnostic batteries for the purpose of validation and calibration of the Japanese version of the SRS.

In regard to the normative sample, questionnaires were distributed by mail to the caregivers of all students attending mainstream classes at primary or secondary schools in the 10 geographical areas making up Japan in 2010 (*n* = 87 548 caregivers). One hundred and forty-eight primary schools and 71 secondary schools participated in this study. All of them were community schools where >93% of children living in the community attend, according to the annual report of Japan's Ministry of Education, Culture, Sports, Science and Technology, 2010 12. Questionnaires were returned for 25 779 children aged 6–15 years (response rate 29.4%). Questionnaires with missing answers were excluded so that all analysis was based on a complete data set, leaving a final normative sample of 22 529 participants (11 455 boys) with SRS data provided by their mothers (*n* = 20 430), fathers (*n* = 1728), both parents (*n* = 166), other caregivers (*n* = 119) or unspecified (*n* = 86). Each of the 9 grade levels comprised a minimum of 754 participants of each sex, and both sexes were proportionally represented (Table 1).

**Table 1:** Social Responiveness Scale total raw score distributions in the normative sample by sex and age (grade)

Grade	Sex				*t*	*P*	*d*

	Males		Females				
	
	N	Mean (SD)	N	Mean (SD)			
1	1655	37.3 (18.2)	1473	33.0 (16.7)	44.3	0.000	0.25
2	1521	36.2 (18.2)	1394	32.1 (16.3)	37.8	0.000	0.24
3	1384	35.4 (19.2)	1432	31.2 (16.4)	39.0	0.000	0.24
4	1375	33.7 (18.4)	1386	30.2 (16.3)	26.2	0.000	0.20
5	1449	33.0 (18.5)	1287	31.0 (17.5)	8.6	0.003	0.11
6	1203	31.9 (19.6)	1229	29.9 (17.8)	6.9	0.009	0.11
7	1072	32.3 (19.1)	1070	30.3 (17.8)	6.7	0.010	0.11
8	1007	32.7 (20.2)	1049	29.8 (18.2)	12.7	0.000	0.15
9	789	31.7 (20.7)	754	28.9 (18.6)	9.2	0.002	0.14
Total	11 455	34.1 (19.1)	11 074	30.9 (17.2)	13.4	0.000	0.18
Total children	22 529		32.5 (18.3)				

Grade 1 children are usually 6–7 years old. Most grade 1 participants were 7 years old at the time of the survey.

The clinical sample consisted of 257 children diagnosed with ASD (ASD group) and 157 children with psychiatric diagnoses other than ASD (non-ASD group) (Table 2). They were patients who visited one of 10 child psychiatric clinics during 2008–2010 and whose caregivers gave informed consent to participate in this study. Their existing clinical diagnoses were confirmed according to DSM-IV-TR criteria 3 based on all of the clinical information available to our research team, which included experienced child psychiatrists and licensed clinical psychologists. Among the 257 children of the ASD group, 229 were subcategorized with 100% diagnostic agreement: 96 with autistic disorder, 65 with Asperger's disorder, 68 with PDD-NOS, and 28 were unspecified. Children in the non-ASD group were diagnosed with adjustment disorder, attention deficit hyperactivity disorder, anxiety disorder, eating disorder, schizophrenia, somatoform disorder, conduct disorder, mood disorder, or mental retardation. Moreover, 61 children recruited from local communities comprised a TD group and were confirmed in diagnostic interviews with the children and their parents to have no history of neuropsychiatric conditions.

**Table 2:** Comparison of Social Responsiveness Scale total raw score between the United States and Japan

Grade	Country				*t*	*P*	*d*

	Japan		US				
	
	N	Mean (SD)	N	Mean (SD)			
1	3102	35.3 (17.6)	71	29.6 (25.6)	1.87	0.06	0.318
2	2891	34.2 (17.4)	92	34.9 (26.9)	0.25	0.80	0.041
3	2786	33.2 (18.0)	109	35.7 (26.8)	0.97	0.33	0.136
4	2739	31.9 (17.5)	227	35.3 (24.9)	2.02	0.04	0.188
5	2703	32.0 (18.0)	214	34.5 (25.3)	1.42	0.16	0.134
6	2408	30.8 (18.7)	211	31.7 (21.5)	0.59	0.56	0.049
7	2123	31.3 (18.4)	161	31.1 (20.6)	0.12	0.90	0.008
8	2040	31.1 (19.1)	137	31.9 (23.7)	0.39	0.70	0.040
9	1532	30.2 (19.7)	124	38.9 (29.2)	3.26	0.00	0.422
Total	22 344	32.5 (18.2)	1626*	33.6 (24.7)	1.76	0.08	0.051

Grade 1 children are usually 6–7 years old. Most grade 1 participants were 7 years old at the time of the survey.

* US data were cited from the SRS manual (p. 28) (10.

The intellectual levels of the children in the clinical sample ranged from normal intelligence to severe mental retardation based on cognitive testing carried out at clinics [various versions of the Wechsler Intelligence Scale and the Revised Kyoto Scale of Psychological Development 13] or educational/administrative records. The proportions of children with normal intelligence in the ASD and non-ASD groups were not significantly different (*χ*^2^ = 1.42, n.s.).

### Measures

#### The social responsiveness scale

The SRS 11 is a 65-item questionnaire of autistic traits for use with 4- to 18-year-olds that can be completed in 15 min by any adult who has observed the child over time in naturalistic social settings. The SRS was developed to assess autistic symptoms or quantitative traits and has subsequently undergone extensive validation in US samples for use in subclinical and clinical child populations^4^, ^14^–^17^ as well as in general child populations for behavioral genetic research 18–20. It also demonstrated satisfactory internal consistency (Cronbach's α > 0.95), inter-rater reliability between parents and teachers (*r* = 0.78, *P* < 0.01), and concurrent validity with an interview-based instrument^21^ (*r* = 0.86, *P* < 0.05 for preschoolers; *r* = 0.48, *P* < 0.05 for children aged 7–12; *r* = 0.77, *P* < 0.001 for adolescents aged 13–18) for Japanese children^22^, ^23^ and also for German children^24^. The Japanese version was used in this study. Higher scores on the SRS indicate higher degrees of social impairment. The 65 SRS items were further categorized into five treatment subscales (social awareness, social cognition, social communication, social motivation, autistic mannerisms) 11. The SRS total scores are generally unrelated to IQ in the normal range and distinguish children with ASD from those with other types of psychopathology 16.

#### The autism diagnostic interview-revised

The Autism Diagnostic Interview-Revised (ADI-R) 25 is a parent-report interview and is a research standard for establishing a diagnosis of autism. To meet the ADI-R criteria for autism, the cutoff must be reached in each domain of reciprocal social interaction, communication, and restricted, repetitive, and stereotyped patterns of behavior. The Japanese version of the ADI-R was used in this study, which has demonstrated good reliability and validity for Japanese children 26.

### Ethical issues

The study protocol was approved by the Ethics Committee of the National Center of Neurology and Psychiatry, Japan. Written informed consent to participate was obtained from the caregivers of each child participant.

### Data analysis

Following examination of the SRS distribution as a function of age and sex, a cross-cultural comparison of SRS total scores provided by parents was performed between previously reported US norms (the SRS manual, p. 28) 11 and the obtained Japanese scores using *t*-tests. Factor analysis was performed using principal components analysis (PCA) on children in the ASD, non-ASD, and TD groups, and the most parsimonious model was subsequently examined by confirmatory factor analysis (CFA) in the normative sample. To address discriminant validity, comparisons of the SRS scores across diagnostic groups were made using analysis of variance (anova) methods with Bonferroni correction whenever appropriate. Intraclass correlation coefficient (ICC) was computed for associations between SRS scores, full scale IQ, and ADI-R algorithm scores. In addition, a receiver operating characteristics (ROC) analysis was conducted to determine the cutoff points for primary and secondary screening; for the former, the cutoff point was where the sum of sensitivity and specificity was the largest, and for the latter, it was where the likelihood was the largest for children in the ASD, non-ASD, and TD groups, for boys and girls separately. Analysis was performed using spss 18.0j for Windows (SPSS Japan Inc., Tokyo, Japan), with amos 17.0j for Windows (SPSS Japan Inc., Tokyo, Japan) used for the confirmatory factor analysis.

## Results

### Population distribution

**Fig. 1:**
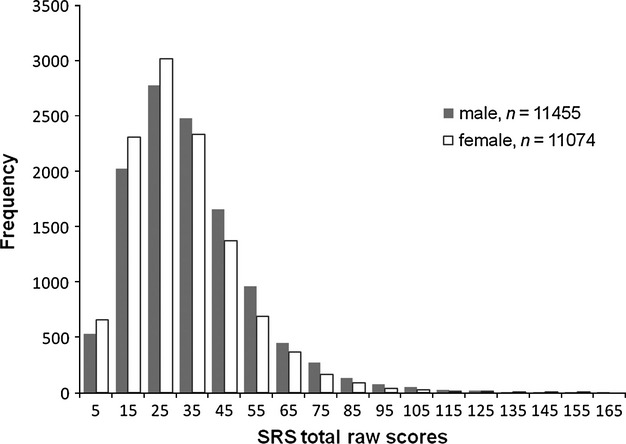
Distribution of Social Responsiveness Scale (SRS) total raw scores rated by caregivers in the general sample of 6- to 15-year-old children.

Social Responsiveness Scale score distribution among 6- to 15-year-old children in the Japanese general population is shown in Fig. 1, and mean SRS total raw scores by age group are presented for boy and girl subsamples in Table 1. To investigate the effects of age (grade) and sex on SRS scores, a 2-way anova (grade × sex) was conducted on the total raw scores. The interaction was significant (*F*_8,180,224_ = 2.00, *P* < 0.05, *η*^2^ = 0.00), and the main effects of grade (*F*_8,180,224_ = 20.03, *P* < 0.001, *η*^2^ = 0.01) and sex (*F*_8,180,224_ = 157.37, *P* < 0.001, *η*^2^ = 0.01) were significant, although the effect size indicates that the differences in the SRS scores by grade and sex were modest.

Mean SRS score of each age group was within 0.2 standard deviations of the entire sample means for boys and girls respectively (boys 30.3–37.9, girls 27.5–34.3). Boys scored higher than girls across the entire age range, with the maximum sex difference seen for the youngest subgroup at grade 1 (*t* = 44.24, *P* < 0.001, *d* = 0.25). Therefore, we standardized the Japanese version of the SRS on each of the boy and girl subsamples across the age range 27.

Table 2 shows our Japanese normative data together with the original US parent and teacher rating data (the SRS manual, p. 28) 11 derived from five different studies. Japanese children scored similarly to their US counterparts, except those in grades 4 and 9; here, Japanese children had significantly lower mean SRS scores than their US counterparts.

#### Factor structure

PCA suggested a one-factor solution for the 475 children comprising the clinical and TD groups (Table 3). Seven items (items 24, 29, 35, 37, 44, 49, 51) with factor loadings >0.600 represented all three of the DSM-IV-TR criterion domains for autism. When 22 items with factor loadings <0.400 were excluded, the first factor explained 34.8% of variance in SRS scores in this sample, consistent with the original US and German data for child psychiatric patients. When performed with the mean scores of the five treatment subscales, rather than the mean scores of 65 items, PCA gave a one-factor solution accounting for 77.2% in this sample.

**Table 3:** Principal components analysis of social responsiveness scale data

Component	ASD, non-ASD, and TD groups (*n* = 475)		

	Total	% of variance	Cumulative%
1	18.928	29.120	29.120
2	3.851	5.925	35.045
3	3.152	4.850	39.895
4	1.926	2.963	42.858
5	1.701	2.616	45.474

ASD, autism spectrum disorders; TD, typical development.

The clinical sample consisted of participants with ASD (*n* = 257) and non-ASD (*n* = 157).

Next, the single-factor model suggested by PCA and by extensive prior research on the SRS 20, 24 was subjected to CFA using data from the normative sample. The comparative fit index, the goodness of fit index, the adjusted goodness of fit index, and root mean square error of approximation were 0.677, 0.739, 0.722, and 0.055 for all 65 items, 0.811, 0.854, 0.840, and 0.055 for 43 items with factor loadings >0.400 derived from PCA on the exploratory set, and 0.989, 0.987, 0.962, and 0.083 for the five treatment subscales. These findings lend support to the notion of a unitary factor influencing the multiple aspects of dysfunction that characterize autistic symptomatology in children in the general population.

### Other psychometric properties

**Fig. 2:**
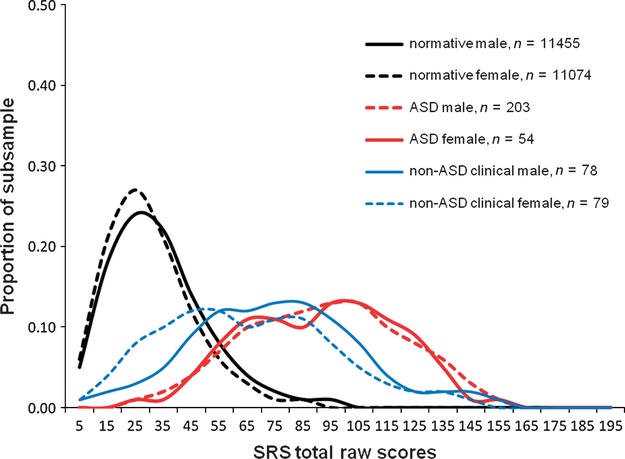
Distribution of Social Responsiveness Scale (SRS) total raw scores in child psychiatric patients with and without autistic spectrum disorders (ASD).

Table 4 indicated that the mean SRS total score of the ASD group was significantly higher than that of the clinical non-ASD (boys *t* = 4.87, *P* < 0.001, *d* = 0.65, girls *t* = 4.68, *P* < 0.001, *d* = 0.83) and TD (boys *t* = 11.73, *P* < 0.001, *d* = 2.29, girls *t* = 11.80, *P* < 0.001, *d* = 2.66) groups. The differences in SRS score were not pronounced among the ASD subcategories: the score did not discriminate between Asperger's disorder and PDD-NOS for either sex, as previously reported 23. As shown in Fig. 2, the SRS scores of both ASD and non-ASD groups were distributed widely and with significant overlap with the general population distribution. Table 5 shows the raw score cutoffs for the 99th, 97.5th, 95th, and 90th percentile values by sex for our normative sample and the proportion of boys and girls with diagnosed ASD who fell within the respective percentile cutoffs. In general, a higher proportion of diagnosed females were at the more extreme percentile rankings in comparison with males.

**Table 4:** Social Responsiveness Scale total raw score means of the ASD, non-ASD, and TD groups

				ASD subcategory			
				
	ASD	nonASD	TD	Autism	Asperger's disorder	PDD-NOS	Unspecified
*N* (Male/Female)	257 (203 : 54)	157 (78 : 79)	61 (30: 31)	96 (77 : 19)	65 (48 : 17)	68 (54 : 14)	28 (24 : 4)
Age (years)							
Mean (SD) Range	10.0 (3.9) 4–18	12.1 (3.7) 4–18	9.61 (2.5) 6–18	9.0 (4.2) 4–18	10.7 (3.1) 4–17	10.0 (4.1) 4–18	11.68 (3.67) 6–17
Intellecual level (N)							
Normal	181	118	57	57	64	59	1
Borderline	14	9	4	8	1	3	2
Mild MR	10	12	0	5	0	3	2
Moderate MR	7	3	0	2	0	1	4
Severe MR	12	8	0	2	0	0	10
MR (unknown level)	33	7	0	22	0	2	9
SRS Mean (SD) Range							
Males	87.6 (27.4) 15–158†	69.7 (27.9) 13–141†	27.4 (16.6) 6–72†	89.5 (24.0) 48–139‡	82.4 (26.8) 15–132	78.4 (26.5) 24–144‡	
Females	86.1 (27.9) 21–153§	62.1 (29.9) 12–134§	21.4 (16.2) 2–65§	91.4 (27.2) 21–133	91.0 (31.4) 38–153	74.7 (25.3) 40–114	
Total	87.3 (27.4) 15–158¶	65.9 (29.1) 12–141¶	24.3 (16.5) 2–72¶	89.8 (24.5) 21–139**	84.6 (28.1) 15–153	77.7 (26.1) 24–144**	

SRS, Social Responsiveness Scale; ASD, autism spectrum disorders; TD, typical development; PDD-NOS, pervasive developmental disorder not otherwise specified; MR, mental retardation.

† ASD > non-ASD, TD (*t* = 4.87, *P <* 0.001, *d* = 0.65; *t* = 11.73, *P <* 0.001, *d* = 2.29, respectively), non-ASD > TD (*t* = 7.79, *P <* 0.001, *d* = 1.67).

‡ Autism > PDD-NOS (*t* = 2.48, *P* < 0.05, *d* = 0.44).

§ ASD > non-ASD, TD (*t* = 4.68, *P <* 0.001, *d* = 0.83; *t* = 11.80, *P <* 0.001, *d* = 2.66, respectively), non-ASD > TD (*t* = 7.17, *P <* 0.001, *d* = 1.52).

¶ ASD > non-ASD, TD (*t* = 7.53, *P <* 0.001, *d* = 0.76; *t* = 17.19, *P <* 0.001, *d* = 2.45, respectively), non-ASD > TD (*t* = 10.51, *P <* 0.001, *d* = 1.59).

** Autism > PDD-NOS (*t* = 3.05, *P* < 0.05, *d* = 0.48).

**Table 5:** Proportion of children with autism spectrum disorders (ASD) corresponding to the 99th, 97.5th, 95th, and 90th percentile values among the ASD group of the Japanese clinical sample

Normative sample (*n* = 22 529)			ASD group (*n* = 257)			
	
Percentile value	Raw score cutoff		*N* (%)			
	
	Males	Females	Males (*n* = 203)		Females (*n* = 54)	
≥99	98	87	70	34.5%	28	51.9%
≥97.5	81	73	117	57.6%	36	66.7%
≥95	70	63	147	72.4%	42	77.8%
≥90	58	53	173	85.2%	44	81.5%

**Fig. 3:**
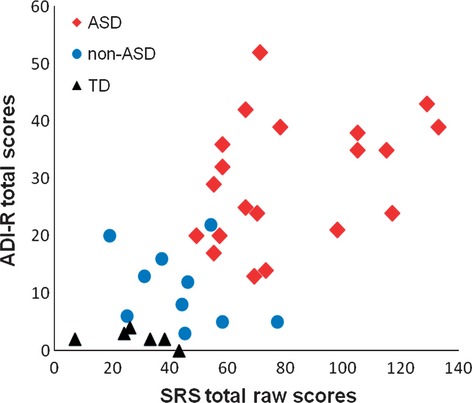
Social Responsiveness Scale (SRS) total raw scores as a function of Autism Diagnostic Interview-Revised (ADI-R) total scores for children with autism spectrum disorders (ASD), non-ASD, and typical development (TD).

Social Responsiveness Scale score did not correlate with IQ (ICC *=* −0.23, n.s.) for 118 participants with IQs > 70 for whom formal test data were available (ASD 46, non-ASD 11, TD 61), although the subgroup with mental retardation tended to score higher. With regard to autistic symptoms, SRS score was significantly correlated with ADI-R total score (ICC = 0.66, *P* < 0.001; Fig. 3), as well as scores for the social interaction domain (ICC = 0.68, *P* < 0.001), communication domain (ICC = 0.58, *P* < 0.001), and restricted and repetitive behavior domain (ICC = 0.50, *P* < 0.001) for a subsample for whom data from both the SRS and ADI-R were available (*n* = 36; ASD 20, non-ASD 10, TD 6; mean age 8.0 years, range 4–18 years).

Receiver operating characteristics analysis informed two sets of cutoff points depending on the purpose of use. When used for primary screening of the general child population such as at school entrance, an optimal cutoff point was 53.5 for boys (sensitivity 0.91, specificity 0.48) and 52.5 for girls (sensitivity 0.89, specificity 0.41). For secondary screening of children referred to clinical settings, where a much higher rate of ASD is expected, the cutoff point of 109.5 for boys (sensitivity 0.23, specificity 0.96, likelihood ratio 6.14) and 102.5 for girls (sensitivity 0.32, specificity 0.95, likelihood ratio 5.73) increases the positive predictive value for ASD diagnosis up to 80.4% for boys and 79.2% for girls, given that the prevalence in Japanese child psychiatric clinics is 40%. Primary and secondary screening cutoffs correspond to a SRS *T*-score of 60 and 90 for boys and 62 and 92 for girls respectively.

## Discussion

We conclude from these data involving a nationwide representative sample of schoolchildren that autistic traits measured by the Japanese version of the SRS are distributed continuously in the population; that the clinical validity of the measurements (in essence, their relevance to autism) appeared strong; and that the findings of this cross-cultural study recapitulate and extend what has been observed in smaller epidemiologic studies of autistic traits in other countries.

The results of this study of quantitative autistic traits – the largest of its kind – add substantial evidence in support of the continuous nature of autistic traits in the general population. This does not mean that individual cases of autism are never discretely or categorically determined. It has long been known, for example, that there exist categorical, relatively rare causes of autistic syndromes (e.g., fragile X syndrome, Rett syndrome, and tuberous sclerosis) caused by single gene abnormalities. The notion of an autistic continuum remains consistent with the existence of such discrete entities. The same is true for mild to moderate intellectual disability, which constitutes the extreme end of a normal distribution (the so-called ‘bell curve’) but comprises a number of discrete syndromes (including but not limited to Down syndrome, Fragile X syndrome, etc.) in the severe end of the symptom distribution. Similarly, segments of the autistic continuum may be comprised of small clusters of discrete disorders (e.g., SHANK 1 mutations, 15q duplications, 16p11.2 deletions) that contribute to intervals at the pathological end of the distribution (for example 75–85, 90–110), but overlap in severity with other cases that represent quantitative accumulations of inherited liability transmitted by polygenic mechanisms or by gene–environment interactions. The causes of cases represented by any given score in the distribution may be independent, partially overlapping, or fully overlapping with the underlying causes of other cases at the same level of severity. The result is a continuous distribution encompassing both discrete and quantitative pathways to affectation across a wide range of severity 28–32. We note that in a recent large general population twin study, Robinson et al. 33 demonstrated overlap in causal influence on autistic symptomatology at each of the first, second, and fifth percentiles of severity in the population.

In our study, there was no evidence of a natural cutoff that differentiated children categorically affected from those unaffected by ASD. The parent-report Japanese SRS cutoff scores for secondary screening derived from our ROC analysis, 109.5 for boys and 102.5 for girls, would comprise approximately 0.5% of our normative sample. On the other hand, the ASD primary screening cutoff with the highest sensitivity, 53.5 for boys and 52.5 for girls, encompassing 10.9% of our normative sample, identifies subthreshold conditions in children that might warrant clinical attention 11. Taken together, these findings complement a recent Korean study 10, in which categorical screening and diagnostic confirmation revealed (and validated) what a continuous distribution of symptom counts. In our normative sample, a parent-report Japanese SRS raw score of 74 for boys and 80 for girls would cut off approximately 3.74%, 1.47% of each gender-specific population distribution, which is very near the prevalence for ASD reported in the Korean study (2.64%) 10.

Our observation of higher quantitative autistic trait scores in males than in females confirms across cultures a subtle but statistically robust gender difference 11, 18, 24. The sex distribution pattern has potentially profound implications for sex disparities universally observed at the extreme end of the distribution (i.e., in clinical ASD cases), where such disparities would be expected to be accentuated, as is true for any normally distributed trait such as height. The magnitude of the sex difference in our sample (*d* = 0.18) was smaller than that in the US data set 11 (*d* = 0.37) but similar to the German normative sample 24 (*d* = 0.16). Accentuation of the gender difference in the US data set could potentially relate to its being derived from a twin sample, given that male twins score higher than non-twins 34. Japanese children diagnosed with ASD were rated as having somewhat lower quantitative trait scores than their US and German counterparts. Such cross-cultural differences could be partly explained by cultural differences in responding to Likert-type rating, on which Japanese informants have a higher tendency to use the midpoint on the scales and US informants a higher tendency to use the extreme values 35.

The results of the exploratory factor analysis for the clinical sample replicate those of previous studies 17, 18, and the results of the confirmatory factor analysis for a very large general population underscore the presence of a primary underlying factor that influences the symptoms representing all three DSM-IV-TR criterion domains of autism. Factor structure has important implications for understanding the core neuropsychological mechanisms underlying autistic traits and symptoms, which are relevant to not only the pursuit of biomarkers and genetic susceptibility factors related to ASD but also diagnostic paradigms 20, 31.

There are two major limitations in this study. First, the response rate was low (29%), although it is keeping with what is expected from population-based surveys. Second, high-scoring children in 22 529 Japanese schoolchildren were not confirmed using any diagnostic instruments, although quantitatively measured autistic traits were extensively clinically confirmed for the separate smaller sample.

In the present study, although the instrument capably distinguished children diagnosed with ASD from children diagnosed with other psychiatric conditions, the score distribution for both clinical groups overlapped. A possible interpretation of this observation, given that autistic traits exhibit considerable independence in causation from many forms of psychopathology in genetic epidemiologic research 15, 36, is that autistic traits, when present, exacerbate other types of psychopathology when they cooccur with autistic traits as comorbid conditions. For some neurodevelopmental conditions, however, it has also become increasingly clear that there are elements of genetic causation that genuinely overlap with the genetic cause of autism; these include ADHD, tic disorders, and developmental coordination disorders, among others 37.

In conclusion, our study provides strong evidence of the continuous nature of autistic symptomatology in the general population, as has been reported in previous studies 1, 18, 19, 37. The findings underscore the notion that paradigms for categorical case assignment are superimposed on a continuous distribution, which can result in substantial variation in prevalence estimation, especially when the measurements used in case assignment are not standardized for a given population (i.e. by gender, informant, culture, etc.). In other words, these data illustrate that when imposing an arbitrary, non-standardized cutoff for diagnosis, small, clinically insignificant changes in the cutoff value can result in significant changes in prevalence, especially when operating at the steeper slopes of the distribution. Our results support the importance, validity, and feasibility of determining standardized quantitative ratings of autistic traits and symptoms across cultures, the implementation of which has the potential to advance international collaborative research on autism and related conditions. Finally, these results call for a rational approach to revising systems of diagnosis and service delivery that currently perpetuate the notion of discontinuity between ASD-affected and unaffected populations.
